# Risk factors and mechanisms of stroke in young adults: The FUTURE study

**DOI:** 10.1177/0271678X17707138

**Published:** 2017-05-23

**Authors:** Mayte E van Alebeek, Renate M Arntz, Merel S Ekker, Nathalie E Synhaeve, Noortje AMM Maaijwee, Hennie Schoonderwaldt, Maureen J van der Vlugt, Ewoud J van Dijk, Loes CA Rutten-Jacobs, Frank-Erik de Leeuw

**Affiliations:** 1 Department of Neurology, Center for Neuroscience, Radboudumc, Donders Institute for Brain, Cognition and Behaviour, Nijmegen, The Netherlands; 2 Department of Neurology, Elisabeth Tweesteden Hospital, PO Box 90151, 5000, LC Tilburg, the Netherlands; 3 Center for Neurology and Neurorehabilitation, Luzern State Hospital, Luzern, Switzerland; 4 Department of Cardiology, Radboudumc, HB Nijmegen, the Netherlands; 5 Department of Clinical Neurosciences, University of Cambridge, Cambridge, UK

**Keywords:** Etiology, ischemic stroke, risk factors, transient ischemic attack, young stroke

## Abstract

Incidence of ischemic stroke and transient ischemic attack in young adults is rising. However, etiology remains unknown in 30–40% of these patients when current classification systems designed for the elderly are used. Our aim was to identify risk factors according to a pediatric approach, which might lead to both better identification of risk factors and provide a stepping stone for the understanding of disease mechanism, particularly in patients currently classified as “unknown etiology”. Risk factors of 656 young stroke patients (aged 18–50) of the FUTURE study were categorized according to the “International Pediatric Stroke Study” (IPSS), with stratification on gender, age and stroke of “unknown etiology”. Categorization of risk factors into ≥1 IPSS category was possible in 94% of young stroke patients. Chronic systemic conditions were more present in patients aged <35 compared to patients ≥35 (32.6% vs. 15.6%, *p* < 0.05). Among 226 patients classified as “stroke of unknown etiology” using TOAST, we found risk factors in 199 patients (88%) with the IPSS approach. We identified multiple risk factors linked to other mechanisms of stroke in the young than in the elderly*.* This can be a valuable starting point to develop an etiologic classification system specifically designed for young stroke patients.

## Introduction

The incidence of ischemic stroke among young adults (18–50 years) is rising and is currently estimated to constitute up to 15–18% of all ischemic strokes.^1,2^ These young individuals, who are often in a period of life during which important decisions on starting a family or a career are being made, remain at high risk for recurrent stroke.^3^ As these patients generally still have a life expectancy of decades ahead, knowledge of risk factors and causes of stroke is essential to inform them on the cause of the disease and to possibly prevent future vascular disease. Despite the ever increasing number of young stroke patients, the risk factors and causes of stroke remain unknown in about one-third of all patients,^4^ partly because of the tendency to classify them according to classification systems developed for elderly stroke patients, for example the TOAST classification.^5^ This classification does not take into account other potential mechanisms of stroke, that particularly occur in the young, including (reversible) vasoconstriction, migraine and non-atherosclerotic (e.g. inflammatory) arteriopathies, as these are seldom causing stroke in elderly patients.^6^ At best, young patients with these causes end up as being classified with a stroke due to an “other determined” cause.

Another potential disadvantage of using a classification system developed for elderly patients is that treatment options based on this approach usually result in an “one size fits all” strategy, usually directed towards the prevention of recurrent thrombus formation, whereas this mechanism might only apply to a part of the young stroke patients.

Conversely, stroke at the other end of the age spectrum, namely between 1 and 18 years, the so-called pediatric stroke, is clearly recognized to be different from stroke in older patients, and risk factors are identified accordingly.^7,8^ Applying this approach to a large group of young stroke patients may lead to an improved identification of risk factors and causes for stroke in young adults, leaving a smaller residual category of patients with an unknown cause of stroke.

Therefore, the aim of this study was to investigate the prevalence of all potential risk factors in patients with a first-ever ischemic stroke or transient ischemic attack (TIA) aged 18–50 years, and categorize them according to the approach of the International Pediatric Stroke Study (IPSS).^7^ The second aim was to evaluate the effect of this approach on the residual proportion of patients that were classified as having an “unknown etiology” according to TOAST criteria. Finally, we aimed to assess whether risk factor categorization according to IPSS may result in identifying more patients with a risk factor or cause of the disease in specific subgroups, such as age or sex.

## Materials and methods

### Study population

This study is part of the FUTURE study (Follow-Up of Transient ischemic attack and stroke patients and Unelucidated Risk factor Evaluation), a prospective cohort study on patients with a stroke at young age. Extensive details of the study have been described previously.^9^ The Medical Review Ethics Committee region Arnhem-Nijmegen approved the study, performed according to the Helsinki Declaration of 1975 (and as revised in 1983). All patients signed informed consent. In short, all consecutive patients aged 18–50 years admitted to our University hospital between 1980 and 2010 with either an ischemic or hemorrhagic stroke or TIA were included. For the present study, we only included patients with a first-ever ischemic stroke or TIA. Exclusion criteria were cerebral venous sinus thrombosis and retinal infarction. TIA was defined as a rapidly evolving focal neurological deficit, without positive phenomena such as twitches, jerks, or myoclonus, with vascular cause only and persisting for a period of less than 24 h, diagnosed by a neurologist.^10,11^ Stroke was defined as a focal neurologic deficit persisting for more than 24 h. Distinction between ischemic and hemorrhagic stroke was made based on radiological findings. From all patients, baseline characteristics were collected. Furthermore, severity of stroke (NIHSS; National Institutes of Health Stroke Scale)^12^ was assessed by a previously validated approach.^13,14^

### Risk factor categorization following IPSS definitions^7^

Information with respect to risk factors (following IPSS definitions), concurrent diseases as well as classical cardiovascular risk factors were extracted from medical charts in a structured, standardized way. Risk factors (either present in patients’ medical history or diagnosed when admitted for stroke) were divided into nine categories, based on IPSS definitions for pediatric stroke: arteriopathy, cardiac disorders, chronic systemic conditions, prothrombotic states, acute systemic conditions, chronic head and neck disorders, acute head and neck disorders, infection and risk factors for atherosclerosis in adulthood (which we changed in “risk factors for early atherosclerosis”). We additionally added one category called “pregnancy”, as pregnancy and the postpartum period (<6 weeks) are one of the most prevalent risk factors in female young stroke patients.^6,15^ Definitions of all categories are found in Table 1. Patients with more than one risk factor or cause were not mutually exclusive to one category.

**Table 1.** Methods: definitions according to the categorization of the International Pediatric Stroke Study (IPSS).

Risk factor category	Definition
1. Arteriopathy	Any arterial abnormality on vascular imaging besides isolated vessel occlusion. Arterial dissection had to be confirmed by angiography (MRA/CTA/conventional)
2. Cardiac disorders	Either a history of chronic cardiac disorder or when detected on ECG or echocardiography during analysis of stroke
3. Chronic Systemic conditions	A condition or disease with known changes in coagulation or vascular structure, such as connective tissue disease, genetic disorder, hematological, inflammatory or immune system disorder, oncological disease, and use of oral contraceptives
4. Prothrombotic states	A known disease in coagulation or found on laboratory testing, such as Factor V Leiden, antiphopholipid syndrome, protein C/S deficiency
5. Acute systemic disorders	Any acute condition that leads to systemic disturbances, e.g. sepsis, hypotension, shock, <72 h after surgery
6. Chronic Head and neck disorders	A disease localized in the area of the head or neck, e.g. migraine, tumor, aneurysm or AVM
7. Acute head and neck disorders	An acute disease, surgery or trauma localized in the head or neck region
8. Pregnancy related^a^	Stroke or TIA during pregnancy or the postpartum period (defined as within 6 weeks after delivery)
9. ≥1 risk factors for early atherosclerosis Separate risk factors were defined as follows^b^	Either a history of a risk factor (mentioned in medical history or the use of medication) or detected during admission or analysis of the stroke in the outpatient clinic. -Diabetes mellitus as a random blood glucose level greater than 200 mg/dL (11.1 mmol/l) or two consecutive fasting venous plasma glucose levels of 126.1 mg/dL (7.0 mmol/l) or greater -Hypertension as systolic blood pressure 135 mm Hg or greater, diastolic blood pressure 85 mm Hg or greater, or both, measured after the first week of the index event -Smoking as at least one cigarette per day in the year prior to the event -Excess alcohol consumption as consuming more than 200 g of pure alcohol per week -Dyslipidemia as a cholesterole level of ≥5.0 mmol/l, LDL of ≥2.5 mmol/l and/or triglycerides of ≥2.0 mmol/l

MRA: magnetic resonance angiography, CTA: computed tomography angiography, AVM: arteriovenous malformation.

a Not present in original IPSS criteria, added for young adults.^6,15^

b Based on definitions of classical risk factors described earlier.^37^

### Classification following TOAST criteria

Assessment of etiology was based on the modified Trial of Org 10172 in Acute Stroke Treatment classification as described earlier.^16,17^ The modified TOAST classification has an additional category: “likely large-artery atherosclerosis.”

### Statistical analysis

Prevalence of risk factors according to the IPSS score was determined. The distribution among the nine categories of the IPSS score was subsequently stratified by age (from 18 to 35 years and from 35 to 50 years), stroke subtype (TIA or ischemic stroke) and sex. In addition, stroke etiology was also categorized by TOAST-classification and stratified by age groups (aged above or under 35 years). Finally, for patients with an unknown cause of stroke according to the TOAST-classification, we determined the number of risk factors according to the IPSS categories. Numbers were presented as means or as medians for data with a normal or non-normal distribution, respectively. For comparison of categorical variables between groups, Chi-square or Fisher’s exact test was used when appropriate. Analyses were performed using IBM SPSS Statistics for Windows version 22.0 (IBM Corp., Armonk, NY, USA). All *p*-values <0.05 were considered significant.

## Results

### Baseline characteristics

Of 1005 patients enrolled in the FUTURE study, 656 cases with an ischemic stroke or TIA were eligible for assessment (Supplemental Figure 1). There was no significant difference between participants and non-participants, except that participants were slightly older than non-participants (40.7 years (SD 7.7) vs. 39.1 years (SD 8.2), respectively). In our cohort, >95% of the patients were Caucasian. The baseline characteristics are shown in Table 2.

**Table 2.** Baseline characteristics of 656 young ischemic stroke or TIA patients.

Characteristic No. (%)	Total 656 (100)	TIA 209 (31.9)	Ischemic stroke 447 (68.1)
Mean age at stroke, yrs [SD]	40.7 [7.7]	40.6 [8.0]	40.8 [7.6]
Age distribution, *n* (%)			
<35 years	138 (21.0)	52 (24.9)	86 (19.2)
≥35 years	518 (79.0)	157 (75.1)	361 (80.8)
Men, *n* (%)	309 (47.1)	94 (45.0)	215 (48.1)
Median NIHSS at admission (IQR)^a^	3 (1–7)	0 (0–1)	5 (2–10)
mRS ≥ 2 at discharge, *n* (%)	140 (21.3)	5 (2.4)	135 (30.2)
Etiology based on TOAST, *n* (%)			
Large artery disease	64 (9.8)	12 (5.7)	52 (11.6)
Likely large artery disease	102 (15.5)	35 (16.7)	67 (15.0)
Cardio-embolic stroke	86 (13.1)	27 (12.9)	59 (13.2)
Small vessel disease	65 (13.1)	11 (5.3)	54 (12.1)
Other defined	96 (14.6)	23 (11.0)	73 (16.3)
Multiple causes	17 (2.6)	2 (1.0)	15 (3.4)
Unknown cause	226 (34.5)	99 (47.4)	127 (28.4)
Period of inclusion, *n* (%)			
1980–1989	143 (21.8)	39 (18.7)	104 (23.3)
1990–1999	171 (26.1)	34 (16.3)	137 (30.6)
2000–2010	342 (52.1)	136 (65.1)	206 (46.1)

TIA: transient ischemic attack, NIHSS: national institutes of health stroke scale, IQR: interquartile range, mRS: modified ranking scale, TOAST: Trial of Org 10172 in Acute Stroke Treatment.

a NIHSS was missing in three cases.

### Categorization of risk factors according to IPSS criteria

**Figure 1.**
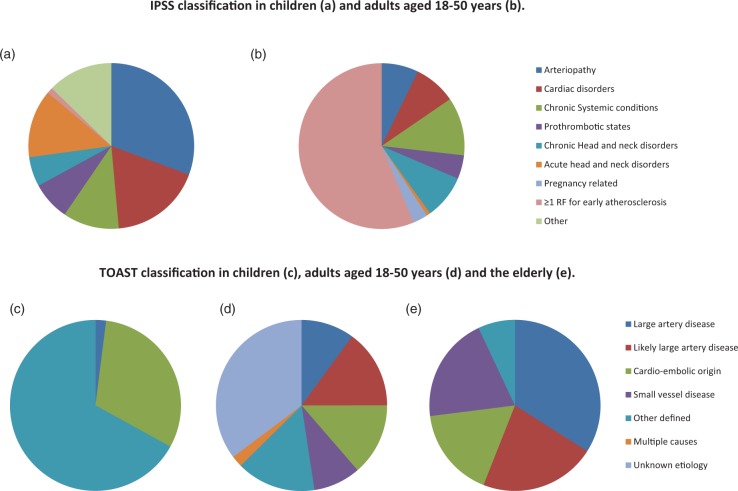
(a–e) Comparison of IPSS versus TOAST classification. Distribution of IPSS risk factors in children <18 years (a) and in young adults aged 18–50 years; data from the FUTURE cohort^6^ (b). Estimation of causes in stroke in children <18 years (c), in young adults aged 18–50 years (the FUTURE cohort)^6^ (d), and in adults >50 years^38^ (e) when classified according to the TOAST classification.

Table 3 and Figure 1 show the risk factors present in 656 young stroke patients, categorized by stroke subtype according to IPSS criteria. We were able to classify 619 patients (94%) into at least one IPSS category, and 315 patients (48.0%) were classified in two or more IPSS categories. None of the young patients was categorized into the IPSS category “infection” (one patient with a tonsillar abscess was listed as “acute head and neck disorder”).

**Table 3.** Prevalence of risk factors in 656 young stroke patients categorized based on IPSS methods.

IPSS risk factor category No. (%)	Total 656 (100)	TIA 209 (31.9)	Ischemic stroke 447 (61.7)
Arteriopathy,^a^ *n*/total (%)	45/368 (12.2)	12/109 (11.0)	33/259 (12.7)
Arterial dissection	29	4	25
CADASIL	1	0	1
Moyamoya	5	2	3
Vasculitis	5	4	1
Vasospasm	3	2	1
Unspecified arteriopathy	1	0	1
Other	1	0	1
Cardiac disorders, *n* (%)	91 (13.9)	26 (12.4)	65 (14.5)
Acquired heart disease	4	2	2
Congenital heart disease	9	4	5
Atrial fibrillation	13	3	3
Myocardial inflammation	8	2	5
Prosthetic valve	9	2	6
Myocardial disease	14	4	10
Valve disease	16	2	13
PFO	14	4	10
PFO+^b^	6	1	5
<72 h after cardiac surgery	4	3	1
Intracardiac thrombus	5	1	4
Other	5	2	3
Chronic Systemic conditions, *n* (%)	126 (19.2)	30 (14.4)	96 (21.5)*
Connective tissue disease	3	1	2
Genetic disorder	4	0	3
Hematological disorder	4	1	3
Immune system disorder^c^	21	4	17
Inflammatory disease	2	0	2
Oncological disease	4	1	3
Oral contraceptive pill	97	23	74
Other	1	1	0
Prothrombotic states, *n* (%)	51 (7.8)	13 (6.2)	38 (8.5)
Acquired thrombophilia	1	0	1
Antiphosholipid syndrome	10	1	9
Factor II deficiency	1	0	1
Factor V Leiden	12	5	7
Hyperhomocysteïnaemia	25	6	19
Increased factor VIII	2	0	2
Protein C/S deficiency	2	1	1
Multiple	3	1	2
Acute systemic disorders, *n* (%)	3 (0.5)	0 (0.0)	3 (0.7)
<72 h after surgery	2	0	2
Hypotension	1	0	1
Chronic Head and neck disorders, *n* (%)	96 (14.6)	37 (17.7)	59 (13.2)
Aneurysm	1	1	0
Brain tumor	3	1	2
Intracranial AVM	1	0	1
MELAS	3	0	3
Migraine	87	35	52
Other cranial tumor	1	0	1
Acute head and neck disorders, *n* (%)	8 (1.2)	3 (1.4)	5 (1.1)
Head or neck surgery	5	2	3
Head or neck trauma	2	1	1
Tonsillar abscess	1	0	1
Pregnancy related, *n*/total (%)	20/347 (5.8)	11/115 (9.6)*	9/232 (3.9)
Post-partum	4	0	4
During pregnancy	16	11	5
≥1 RF for early atherosclerosis^d^, *n*/total (%)	586/615 (95.3)	185/196 (94.4)	401/419 (95.7)
≥2	346 (59.5)	101	245
≥3	119 (19.3)	35	84
≥4	21 (3.2)	5	16

Note: *p*-Values represent the difference between young TIA and Ischemic stroke population. Patients were not exclusive to one category. In some cases, the sum of the subcategories leads to a higher total than presented due to patients who carried more than one risk factor in a category. TIA: transient ischemic attack, CADASIL: cerebral autosomal dominant arteriopathy with subcortical infarcts and leukoencephalopathy, PFO: Patent foramen ovale, MELAS: Mitochondrial encephalomyopathy, lactic acidosis, and stroke-like episodes, RF: risk factor.

a Vascular imaging (MRA/CTA/conventional angiography) not performed in *n* = 288 (excluding duplex of carotid arteries).

b PFO+: Patent foramen ovale in combination with a thrombus, septumaneurysm or left–right shunting.

c Sjogren’s disease (*n* = 1), Wegener’s disease (*n* = 1), systemic lupus erythematosus (SLE, *n* = 13), and immune thrombocytopenic purpura (ITP, *n* = 5).

d Complete information was unknown in *n* = 41.

* *p* < 0.05.

Chronic systemic conditions (19.2%), chronic head and neck disorders (14.6%), cardiac disorders (13.9%) and arteriopathy (12.2%) were the most reported risk factor categories among patients with a young stroke, besides ≥1 risk factors for early atherosclerosis (95.3%).

When stratified according to age (Table 4), we found that chronic systemic conditions were significantly more present in stroke patients <35 years old compared with those ≥35 years old (32.6% vs. 15.6%, *p* < 0.05). On the other hand, risk factors for early atherosclerosis were more present in stroke patients aged 35 or older versus patients younger than 35 years (96.9% and 89.0% respectively, *p* < 0.05).

**Table 4.** Prevalence of IPSS risk factor categories in 656 young stroke patients stratified by age and sex, respectively.

IPSS Risk factor category No. (%)	<35 years 138 (21.1)	≥35 years 518 (78.9)	Men 309 (47.1)	Women 347 (52.9)
Arteriopathy,^a^ *n*/total (%)	10/73 (13.7)	35/295 (11.9)	17 (5.5)	28 (8.1)
Cardiac disorders, *n* (%)	18 (13.0)	73 (14.1)	35 (11.3)	56 (16.1)
Chronic systemic conditions, *n* (%)	45 (32.6)*	81 (15.6)	12 (3.9)	114 (32.9)*
Prothrombotic states, *n* (%)	15 (10.9)	36 (6.9)	18 (5.8)	33 (9.5)
Chronic head and neck disorders, *n* (%)	23 (16.7)	73 (14.1)	26 (8.4)	70 (20.2)*
Acute head and neck disorders, *n* (%)	4 (2.9)	4 (0.8)	5 (1.6)	3 (0.9)
Pregnancy related,^b^ *n*/total (%)	15/94 (16.0)*	5/253 (2.0)	N/A	20 (5.8)
≥1 RF for early atherosclerosis,^c^ *n*/total (%)	113/127 (89.0)	473/488 (96.9)*	277/288 (96.2)	309/327 (94.5)

Note: The category “acute systemic disorders” was not incorporated because of small numbers (*n* = 3). *p*-Values represent the difference between young stroke patients aged <35 years versus ≥35 years or sex, respectively. RF: risk factor.

a Vascular imaging (MRA/CTA/conventional angiography) not performed in *n* = 288 (excluding duplex of carotid arteries).

b Women, *n* = 347

c Complete information was unknown in *n* = 41.

* *p* < 0.05.

When stratified by sex (Table 4), women were more likely to have a chronic head and neck disorder and a chronic systemic condition than men (20.2% vs. 8.4%, *p* < 0.0001; 74.7% vs. 25.3%, *p* < 0.05).

### Classification according to TOAST criteria

The distribution of patients across the various TOAST categories was age-dependent (Table 5). Patients aged ≥35 years were more likely to be classified as having “large artery disease” than patients aged <35 years (11.6% vs. 2.9% *p* < 0.05) or as “likely large artery disease” (18.3% vs. 5.1% *p* < 0.05). On the other hand, stroke was more likely to be classified as “other defined etiology” in patients younger than 35 years (23.2% vs. ≥35 years 12.4% *p* < 0.05). Stroke of cardio-embolic origin was equally found across both age groups (<35 years: 11.6% vs. ≥35 years: 13.5%, *p* < 0.05).

**Table 5.** Classification of 656 young stroke patients based on TOAST criteria stratified by age.

TOAST category No. (%)	<35 years 138 (21.1)	≥35 years 518 (78.9)
Large artery disease, *n* (%)	4 (2.9)	60 (11.6)*
Likely large artery disease, *n* (%)	7 (5.1)	95 (18.3)*
Cardio-embolic origin, *n* (%)	16 (11.6)	70 (13.5)
Small vessel disease, *n* (%)	8 (5.8)	57 (11.0)
Other defined, *n* (%)	32 (23.2)*	64 (12.4)
Multiple causes, *n* (%)	3 (2.2)	14 (2.7)
Unknown etiology, *n* (%)	68 (49.3)*	158 (30.5)

Note: *p*-Values represent the difference between young stroke patients <35 years versus ≥35 years. TOAST: Trial of Org 10172 in Acute Stroke Treatment.

* *p* < 0.05.

In 226 patients (34.5%), the cause of stroke was not found according to TOAST criteria and was accordingly classified as “unknown etiology” (Table 6 and Figure 1); this was the most reported category in patients younger than 35 years (49.3% vs. ≥35 years 30.5%, *p* < 0.05).

**Table 6.** Prevalence of risk factor categories based on IPSS in the group of patients classified as “stroke of unknown etiology” using TOAST criteria.

IPSS Risk factor category No. (%)	Stroke of unknown etiology *n* = 226
Arteriopathy, *n* (%)	0 (0.0)
Cardiac disorders, *n* (%)	1 (0.4)
Chronic Systemic conditions, *n* (%)	44 (19.5)
Prothrombotic states, *n* (%)	14 (6.2)
Acute systemic disorders, *n* (%)	0 (0.0)
Chronic Head and neck disorders, *n* (%)	38 (16.8)
Acute head and neck disorders, *n* (%)	1 (0.4)
Pregnancy related, *n*/total (%)	10/123 (8.1)
≥1 RF for early atherosclerosis, *n*/total (%)	193/209 (92.3)

RF: risk factor.

According to IPSS categorization, we found additional risk factors in 198 out of these 225 patients (88.0%). We identified 13 patients (5.8%) with a prothrombotic disorder, 11 patients (4.9%) with hyperhomocysteinemia, one patient (0.4%) with antiphospholipid syndrome and one patient (0.4%) with Factor V Leiden. Migraine was reported in 38 patients (16.9%). Ten cases (8.1%) were pregnancy related, with nine women suffering from stroke during pregnancy and one woman within six weeks postpartum. We found 44 patients (19.6%) with a chronic systemic condition; three patients (1.3%) with an autoimmune disorder, one patient (0.4%) with a hematological disease, one patient with an active oncological disease and one patient with a genetic disorder. Oral contraceptives were used by 37 women (16.4%). Also, 193 patients (92.3%) out of these 226 with unknown etiology had at least one risk factor for early atherosclerosis. No arteriopathies or cardiac disorders were found.

## Discussion

We have shown that classifying risk factors according to the IPSS in patients with a stroke at young age may lead to a more appropriate categorization of risk factors. By doing so, we were able to find at least one risk factor in 94% of 656 patients. In addition, in almost 90% of patients who would have been categorized as “unknown etiology” according to the TOAST classification we were able to identify risk factors. This calls upon an appropriate stroke classification system specifically tailored for younger stroke patients. To our knowledge, we are the first to apply a pediatric stroke risk factor categorization to a large young stroke population leading to a unique opportunity to characterize stroke or TIA in young adults more accurately.

However, also some limitations need to be addressed. Due to the long inclusion period, diagnostic measures and strategies may have changed over time leading to variable approaches of risk factor identification as well as new etiologic insights might have emerged. This is supported by the fact that we found patients more likely to be classified into a specific category (e.g. arteriopathy, prothrombotic disorders and chronic systemic conditions) when they suffered a stroke more recently (inclusion period 2000–2010 compared with those included between 1980 and 1990).

Second, initially our data were not collected according to the study protocols of the IPSS, thus some specific (pediatric) risk factors were only identified when mentioned in the history of the patient or when a specific situation warranted further diagnostic evaluation (such as genetic disorders). This might potentially have caused some misclassification. Also, in case of short-term survivors (3.4% first month, 5.9% first year), the diagnostic process might not have been performed to full extent. However, we feel that our approach, if any, would only have led to an underestimation of presence of risk factors, although we were able to find a least one risk factor in 94% of patients.

Furthermore, our data cannot directly be used as a mutually exclusive classification system because identification of risk factors is not limited to only one IPSS risk factor category. Also, amongst all reported risk factors, some are qualified as a necessary causal factor for stroke whilst other factors can be considered as sufficient causes.^18^ However, this appears almost to be inevitable with classification systems of causes of stroke as virtually no one witnesses the true origin of the embolus that causes the stroke.

In addition, it is important to recognize that IPSS identifies risk factors that are not necessary causes of stroke. However, this in-depth phenotyping of patients may be a stepping stone for future research in the identification of novel mechanisms and causes of stroke in young adults.

If we compare our data to the results of the IPSS Registry,^7^ among the most frequent reported categories in pediatric stroke were arteriopathy (53%) and cardiac disorders (31%). These percentages point towards the same direction as we found in our young stroke patients, albeit that the percentages were higher in the IPSS registry because of the large proportion of focal cerebral arteriopathies and congenital heart disease in children, respectively. Chronic systemic conditions were equally present in young adults and the pediatric stroke patients described in IPSS cohort (19%).

A large difference though was found for the presence of one or more risk factors for early atherosclerosis in only 2% of children versus more than 90% of young stroke patients. Especially, patients aged 35 years and older at time of stroke were more likely to have risk factors for early atherosclerosis and therefore were more likely to be classified as “(likely) large artery disease” according to TOAST criteria. This is consistent with the current findings that risk factors for atherosclerosis increase with age.^1^ On the other hand, patients younger than 35 years at stroke onset are more likely to be classified as a stroke of “unknown origin,” and this was the case in almost 50% of our patients. Overall, more than one-third (34.5%) of our cohort was left unclassified with the aid of the TOAST criteria, which is comparable with the current literature of 30–40%.^19,20^ Criticism has been raised on this large proportion of unknown etiology and also on the applicability of TOAST classification on a (young) stroke population.^21,22^ Using IPSS, we were able to identify additional risk factors in 88% of patients otherwise classified as “unknown”. These factors are considered to be risk factors in pediatric stroke patients,^9^ but to date have not been implemented in a stroke classification such as TOAST. An important explanation for the large proportion of unclassified stroke in the young is that several *other* mechanisms of stroke not related to known mechanisms such as atherosclerosis or cardioembolic disorders were not considered as a separate category/entity in TOAST, including non-atherosclerotic arteriopathy, changes in the haemostatic balance, and migraine.^23^ In our cohort, we found that at least 12% of patients had a non-atherosclerotic arteriopathy, emphasizing the importance to recognize this category as a separate entity in young stroke patients. Dissection, both traumatic and non-traumatic, was the most prevalent arteriopathy in our cohort, but other non-atherosclerotic diseases such as vasculitis (due to multiple causes) or moyamoya disease were also found. These diseases are thought to cause ischemia by a thrombotic occlusion rather than by emboli derived from atherosclerotic plaques.^24^ In antiphospholipid syndrome, various mechanisms of stroke have been described. First, antibodies binding to antiphospholipids on the endothelial surface are thought to cause a hypercoaguable state.^25^ In addition, cardiac valve vegetations may develop (so called Libman-Sachs endocarditis) that ultimately can cause cardio-embolic stroke.^26^ Also, vasospasm, e.g. due to substance abuse, can lead to ischemia through direct insufficient perfusion.^27^ Another mechanism underlying stroke is a coagulation disorder. In our cohort, at least 8% of all patients carried one or more factors altering the haemostatic balance. This is in line with a recent paper in which it was suggested that hypercoagulability should be taken into account in the diagnostic process after young ischemic stroke.^4^ We did not find patients with genetic coagulation disorders that are known to be overrepresented in certain ethnic groups such as sickle cell anemia or specific arteriopathies (such as moyamoya disease) that more frequently occurs in the Japanese population.^28^

There is still discussion about the mechanism of stroke within the context of migraine. A recent study shows that migraine (especially with aura) should be considered as a risk factor for ischemic stroke and TIA.^29^ For migraine with aura, it is hypothesized that the cortical spreading depression is responsible for hypoperfusion and/or a local inflammatory response.^30^ In addition, genome-wide association studies showed that there is a shared genetic basis between ischemic stroke and migraine.^31^ There is also increasing evidence for a role of a preceding infection and stroke. Both inflammatory responses causing platelet activation and endothelial dysfunction, as well as hypercoaguability due to immobilization and dehydration when having an infection have been proposed as possible pathophysiological theories. The inflammatory response causes increased concentrations of plasma fibrinogen, C-reactive protein (CRP), and interleukin-6 (IL-6), ultimately resulting in a hypercoaguable state. The role of inflammation as a cause of arterial plaque instability is currently being investigated.^32,33^

Pregnancy and the postpartum period are well-known conditions with an increased risk of stroke. There are several explanations for these observations. First, pregnancy-related disorders such as preeclampsia, gestational hypertension, gestational diabetes and on the furthest end of the range, a HELLP-syndrome (Hemolysis, Elevated Liver enzymes Low Platelet count) increase the risk of stroke. Furthermore, pregnancy itself comes with a hypercoaguable state with higher levels of coagulation factors (II, VII, VIII, IX, X, XII en XIII), most pronounced in the third trimester, which is thought to be a protective mechanism of the women’s body to prevent high volumes of blood loss during the delivery.^34,35^ At last, complications during the partus such as traumatic dissection, amniotic fluid emboli and postpartum angiopathy may also cause stroke.^34,36^

To date, current treatment options in young stroke patients are generally focused on the removal of an acute thrombus or prevention of thrombus formation, that perhaps does not even play a role in the etiology of some young stroke patients. Considering all these other mechanisms for stroke from the view of a young adult a “one-size-fits-all” therapy is not effective. In pediatric stroke, this is supported by the fact that to date, a favorable effect of trombolysis could not be demonstrated and that secondary preventive medication is generally not indicated because only a small proportion of all children (in IPSS 2%) carried risk factors for atherosclerosis.^7,24^ The effectiveness of a treatment can be assessed more accurately when clinical trials as well as clinicians could rely on a classification system that takes into account all possible mechanisms of young stroke.

In summary, several other than the “classic” cardiovascular risk factors or cardiac diseases can be found in young stroke patients. We propose that etiology of stroke in young adults should preferably not be classified according to a classification system designed for elderly stroke patients, such as TOAST classification, specifically in patients aged below 35 years of age. Using a risk factor categorization according to IPSS, we were able to identify at least one risk factor in 94% of all young stroke patients. Thereby we were able to find additional risk factors in 88% of patients otherwise classified as “unknown etiology” according to TOAST classification. Therefore, our modified risk factor inventory based on pediatric findings provides a valuable starting point for the development of a young stroke-specific classification system. Future studies are warranted that confirm the causality of these risk factors and to develop and validate an etiologic classification approach specifically for young stroke patients. This ultimately can lead to better treatment strategies focused on the specific mechanism underlying young stroke.

## Supplementary Material

Supplementary material
