# Supplementary figures and images for: Risk factors and mechanisms of stroke in young adults: The FUTURE study
Source: J Cereb Blood Flow Metab. 2017 May 23;38(9):1631–41. doi: 10.1177/0271678X17707138 (PMC6120122; doi:10.1177/0271678X17707138)

Supplemental figure 1. Flow chart of FUTURE study young stroke population.

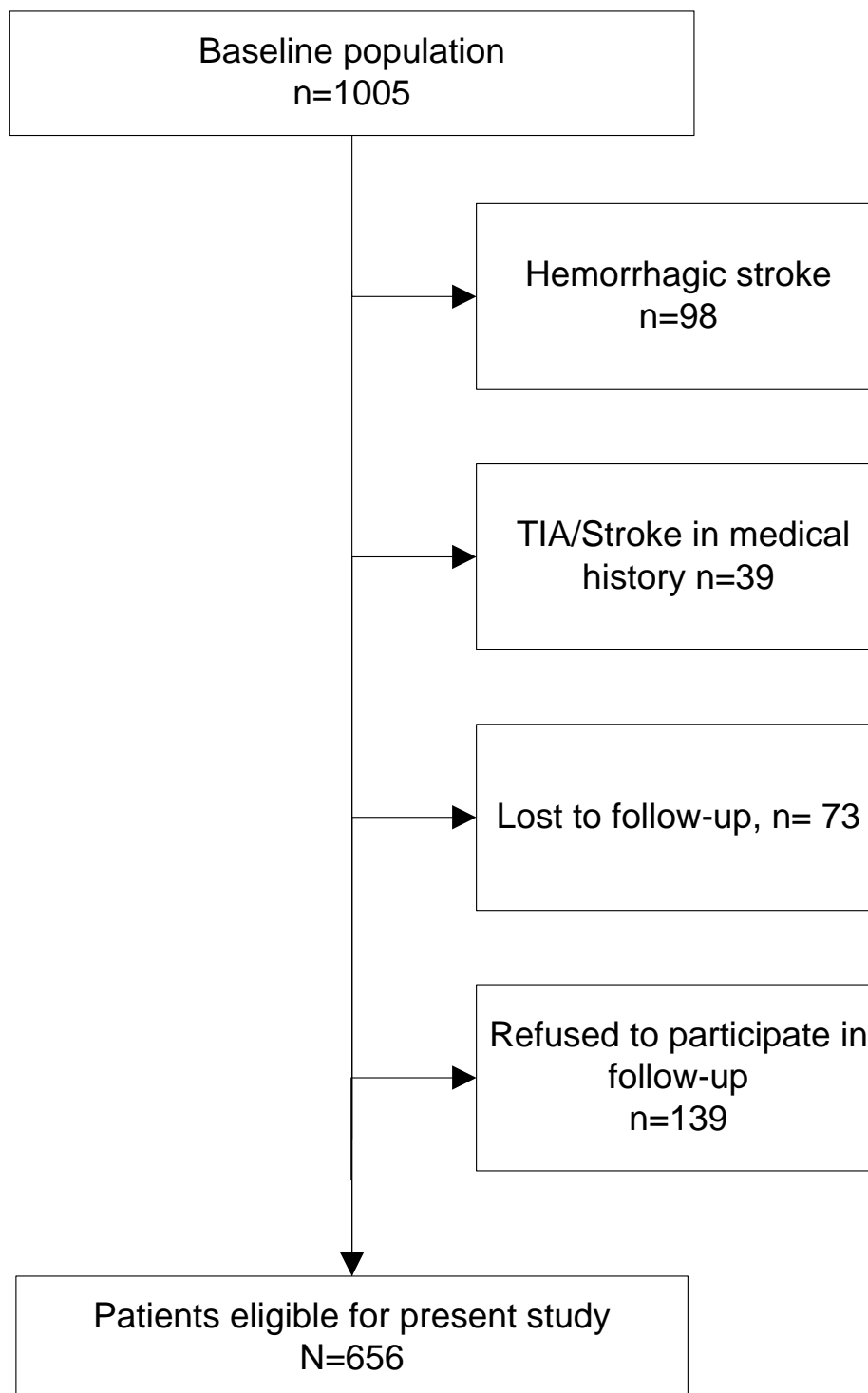

Supplement: Supplementary material [file JCB707138_supplementary_material.pdf]
